# Risk/Benefit Evaluation of Chia Seeds as a New Ingredient in Cereal-Based Foods

**DOI:** 10.3390/ijerph20065114

**Published:** 2023-03-14

**Authors:** Marta Mesías, Pablo Gómez, Elena Olombrada, Francisca Holgado, Francisco J. Morales

**Affiliations:** Institute of Food Science, Technology and Nutrition (ICTAN-CSIC), 28040 Madrid, Spain

**Keywords:** chia (*Salvia hispanica* L.), seeds, biscuits, Maillard reaction, acrylamide, furanic compounds, nutritional properties, risk/benefit, safety, public health

## Abstract

Chia seed (*Salvia hispanica* L.) is a food rich in protein, fiber, polyunsaturated fatty acids and antioxidants. Consequently, its incorporation in food formulations may be desirable from a nutritional and healthy point of view. However, there is concern regarding the formation of process contaminants when they are subjected to thermal processing. The objective of this study was to incorporate different amounts of ground chia seeds in a biscuit model to evaluate the effect on the antioxidant capacity and formation of acrylamide and furfurals. Seven standard “Maria-type” biscuit formulations were prepared, replacing wheat flour with different amounts of ground chia seeds (defatted and non-defatted), from 0% (control biscuit) to 15% (respect to total solids in the recipe). Samples were baked at 180 °C for 22 min. Compared with the control biscuit, chia formulations increased the content of nutrients, antioxidant capacity (ABTS) and phenolic compounds (Folin–Ciocalteau method) but also doubled acrylamide levels and even raised more than 10 times furanic compound concentrations. Results indicate that the use of chia seeds as ingredients in new cereal-based formulations would improve the nutritional profile but also increase the occurrence of chemical process contaminants. This paradox should be carefully considered in the context of risk/benefit analysis.

## 1. Introduction

In the last decades, the diet of the population has been characterized by an excessive intake of saturated fats due to the consumption of fast food or ultra-processed foods, a high consumption of proteins of animal origin and a low consumption of fruits and vegetables, reducing then the fiber intake. These dietary habits, together with sedentary lifestyles, have raised obesity rates and obesity-related diseases, such as diabetes or cardiovascular disease [1]. Considering the relationship between nutrition and health, to deal with these problems, consumers are urged to reduce fat and sugars from their diets whenever possible. The new consumer demands for healthier products have led to a revolution in the food industry, trying to offer healthier and added-value alternatives through the reformulation of their products. These changes have been accentuated by social demands and special nutritional needs, with a high trend to follow vegan, gluten-free or lactose-free diets, among other habits. All these factors make the food industry to be faced the challenge of satisfying a variety of nutritional needs and eating habits by diversifying its product range [2].

An example of innovative foods is the new formulations of cereal-based foods, which comprise a large food category with significant differences in the typology of the finished product according to the recipe and the processing applied. Indeed, innovative formulations with alternative ingredients are growing in consumer preferences. This aspect is evident in the biscuit sector, where the traditional wheat-flour biscuit made of sugars and fats is moving to novel recipes mixed with different grains or including emerging cereals, pseudocereals and seeds such as chia or sesame in order to provide consumers with low-sugar, gluten-free alternatives, high fiber, or mineral-enriched products, among others [3].

Focusing on chia (*Salvia hispanica* L.), this seed is characterized by being rich in polyunsaturated fatty acids (ω-3 fatty acids (linolenic acid, 54–67%) and ω-6 (linoleic acid, 12–21%)) and poor in saturated fatty acids [4]. It also contains proteins (15–24%), dietary fiber (18–30%), carbohydrates (26–41%) and minerals (4–6%) [5]. The protein content of chia is higher than in other traditional crops, i.e., wheat, corn, rice and oats [6], and it has bioactive compounds with high antioxidant activity, such as sterols, tocopherols, carotenoids and phenolic compounds, mainly flavonol glycosides, chlorogenic acid and caffeic acid [2,7]. In addition, chia seed is able to absorb high amounts of water, leading to the formation of a transparent gel called chia mucilage, which is essentially composed of soluble fiber [8]. Due to these properties, chia is considered a functional food for human nutrition with remarkable nutritive characteristics, enabling its application in the food industry as a thickener, emulsifier, stabilizer or antifreeze agent [6,8] as well as a functional ingredient with positive effects on health. For that reason, the incorporation of chia in the formulations of certain foods may be especially desirable both for technological reasons and from a nutritional and healthy point of view. However, in 2019, the European Food Safety Authority (EFSA) stated that the use of chia should be limited to non-thermally treated foods due to the possible formation of chemical process contaminants, such as acrylamide and furanic compounds [9].

In previous work, the acrylamide formation in different formats of chia seeds roasted under different conditions was evaluated, demonstrating an increase dependent on the intensity of heat treatment, the physical integrity of the chia seed and the fat content of seeds [10]. At this point, it is necessary to delve into these aspects and assess the addition of these seeds as ingredients in new food formulations in a risk/benefit context. To this purpose, the objective of this study was to evaluate the incorporation of different quantities and formats of chia seeds in a novel formulation of wheat-based biscuits, considering positive consequences but also food safety aspects associated with the generation of the chemical process contaminants. Results will provide new insights that will contribute to the increase of information about this food matrix and the consequences when it is heat-treated, being limited or inconclusive up to now.

## 2. Materials and Methods

### 2.1. Samples

Black chia seeds (*Salvia hispanica* L.) originating from Mexico and certified for organic cultivation were purchased from a local market (Madrid, Spain) (Lot C-O-AD18-3-V1, importer EcoAndes Import Export, S.L.). Chia seeds were subjected to different physical-chemical treatments before the incorporation into the biscuit formulations. The following samples were obtained: (1) ground chia seed (GS)—whole seeds were ground using an Ultra-Turrax to obtain a homogeneous mixture and passed through a mesh size sieve of 0.80 mm; (2) defatted ground chia seed (DGS)—ground seeds were washed twice adding 500 mL of hexane to 50 g of sample and left to dry for 20 h at room temperature. Wheat flour and other food-grade ingredients were purchased from local supermarkets in Madrid, Spain. The nutritional composition of wheat flour and chia seeds were based on the information declared on the label package. In order to check the reduction of fat in the defatted seeds, the fat content was determined by Soxhlet extraction with petroleum ether as the solvent, according to AOAC 920.39 [11], in both defatted and non-defatted ground chia seeds.

### 2.2. Preparation of Biscuits

Seven biscuit models were prepared according to the recipe described in the AACC (American Association of Cereal Chemists) method 10–54 [12]. The control biscuit was formulated with 100% wheat flour, and experimental biscuits were prepared to replace wheat flour with ground chia seeds and defatted ground chia seeds (percentages in the final weight 5%, 10% and 15%). In this way, the final amount of solids in the dough always remained the same (Table 1).

Ingredients were thoroughly mixed, and the dough was rolled out into disks with a diameter of 6.5 cm and a thickness of 2 mm and baked at 180 °C for 22 min in a conventional oven (Memmert UFE 400, Schwabach, Germany). Twelve biscuits per batch and two batches per formulation were prepared. Three biscuits per batch were ground and mixed and analytical determinations were performed in duplicate for each mixture, thereby obtaining two different values from the two batches corresponding to six different biscuits per formulation. For the determination of hardness, two biscuits per batch for each formulation were used.

### 2.3. Determination of Weight Loss after Baking

The weight of the biscuits was recorded before and after baking for each trial. The percentage of weight loss related to moisture loss was determined as follows:

Weight loss (%) = 100 − [sample weight after baking (g) × 100/sample weight before baking (g)].

### 2.4. Determination of Moisture and Water Activity (Aw)

Moisture was determined gravimetrically in an oven at 105 °C for 24 h according to the AOAC method [13]. The water activity of biscuits was measured at 25 °C by an AquaLAB CX-2 (Decagon Devices Inc., Pullmand, WA, USA).

### 2.5. Measurement of pH

Ingredients and biscuits (1 g) were mixed with 100 mL of water and vortexed for 3 min. The mixture was held at room temperature for 1 h and centrifuged to separate phases. pH of the supernatant was measured using a CG-837 pH meter (Schott, Mainz, Germany).

### 2.6. Determination of Color

The color measurements were made using a portable spectrophotometer (Konica Minolta CM-1600d). Three independent measurements of *a* * (redness), *b* * (yellowness) and *L* * (lightness) parameters were carried out on different areas of both ingredients and biscuit samples. The E index was calculated according to the following equation [14]:

E = (*L*^2^ + *a*^2^ + *b*^2^)^1/2^.

### 2.7. Determination of Hardness

The hardness of the biscuits was evaluated using the Texture Analyzer TA–TXPlus (Texture Technologies Corporation, Scarsdale, NY, USA) equipped with a 50 kg load cell, a probe (Warner–Bratzcer, HDP/BSK knife model) with a compression speed at 1 mm/s and a distance prolongation of 10 mm. The force at the first major drop in the force–deformation curve (Fmax) and deformation at maximum force were obtained for four replicates per batch. The results of hardness were expressed as N (Newton).

### 2.8. Determination of Asparagine

Asparagine content in the samples before and after heating was determined by gas chromatography-flame ionization detection (GC-FID), according to Mesías et al. [15]. A GC-FID (Agilent GC 7820A-FID) equipped with an automatic injector and an amino acid dedicated column (Zebron ZBAAA capillary; 10 × 0.25 mm) was used. The starting oven temperature was set at 110 °C and increased by 32 °C per minute until 320 °C was reached. An aliquot of the derivatized sample (1 μL) was injected in split mode (15:1) at 250 °C. The FID detector was set to 320 °C, and the carrier helium gas flow rate was maintained at 1.5 mL/min whilst in process. External calibration was carried out at five levels (20, 50, 100, 150 and 200 mmol/mL) with the asparagine standard, and results were corrected for recovery with norvaline as the internal standard. Free asparagine content was expressed as mg/100 g of sample.

### 2.9. Determination of Phenolic Acids

Phenolic acids (*p*-hydroxybenzoic acid, syringic acid, vanillic acid, *p*-coumaric acid, caffeic acid, ferulic acid, protocatechuic acid and chlorogenic acid) were determined chromatographically according to the method described by Mesías et al. [16]. Results were expressed as µg/g sample.

### 2.10. Determination of Total Phenolic Content

Total phenolic content (TPC) was determined by the Folin–Ciocalteu method. Direct measurement of the samples without extraction prior analysis was according to Horszwald et al. [17]. The limit of quantitation (LOQ) was set at 0.60 µg/g. Results were expressed as mg gallic acid equivalents (GAE)/g sample.

### 2.11. Total Antioxidant Capacity by Direct ABTS Assay

The direct measurement of the total antioxidant capacity was performed according to Gökmen et al. [18] and adapted to the microplate reader. Trolox was used for the calibration, and results were expressed as µmol equivalents of the trolox (TEAC)/g sample.

### 2.12. Determination of HMF and Furfural

HMF and furfural were determined following the HPLC method described by Mesias et al. [19]. Analysis was carried out using a Shimadzu HPLC system (Kyoto, Japan) equipped with an LC-20AD pump, a SIL-10ADvp autosampler, a CTO-10ASVP oven and an SPDM20A diode array detector. The chromatographic separation was performed on a Mediterranean Sea ODS-2 (250 × 4.0 mm, 5 μm, Tecknokroma, Barcelona, Spain) using two mobile phases: solvent A (0.1% formic acid) and solvent B (acetonitrile) at a flow rate of 1 mL/min in gradient elution mode. The gradient elution started at 5% B until minute 12 and increased linearly to 60% in 1 min, remaining in isocratic until minute 14 and returning to the initial conditions in 1 min. The total run time was 22 min, the diode-array detector was set at 280 nm and 20 μL of the extract was injected. The external standard method with pure standards was used to quantify HMF and furfural. The LOQ was set at 0.6 mg/kg and 0.3 mg/kg for HMF and furfural, respectively. Results are expressed as mg/kg samples.

### 2.13. LC-ESI-MS-MS Determination of Acrylamide

Acrylamide was determined as described by Mesías et al. [19]. An Agilent 1200 liquid chromatograph coupled to an Agilent-G6410B Triple Quadrupole MS detector (Agilent Technologies, Palo Alto, CA, USA) in the positive electrospray ionization was used. The sample (5 µL) was separated onto a Hypercarb (50 × 2.1 mm, 5 mm; Thermo Scientific, Bremen, Germany) at 30 °C with 0.2% formic acid in water at a flow rate of 0.4 mL/min. The mass transitions *m*/*z* 72 > 55 and *m*/*z* 75 > 58 were used for the identification and quantification of acrylamide and the isotope-labeled internal standard [^13^C_3_]-acrylamide, respectively. The mass transitions *m*/*z* 72 > 44, 72 > 27 for acrylamide and *m*/*z* 75 > 44 for the isotope-labeled internal standard [^13^C_3_]-acrylamide were used as qualifiers. The LOQ was set at 15 μg/kg. Results were expressed as μg/kg sample. The recovery rate of acrylamide spiked in the samples was between 90 and 106%. Relative standard deviations (RSD) for the precision, repeatability and reproducibility of the analysis were calculated as 2.9%, 2.1% and 3.3%, respectively. The accuracy of results is demonstrated through the participation in proficiency tests launched by the Food Analysis Performance Assessment Scheme (FAPAS) program. The latest results for the food matrices provided for FAPAS were coffee (test 30117, December 2021), crispbread (test 30118, January 2022) and potato crisps (test 30120, April 2022), yielding z-scores of 0.4, −0.1 and 0.1, respectively.

### 2.14. Statistical Analysis

Statistical analyses were performed using SPSS version 26 (SPSS, Chicago, IL, USA). Data were expressed as mean ± standard deviation (SD). One-way ANOVA followed by Scheffe’s test or a Student’s *t*-test was used to identify the overall significance of differences. All statistical parameters were evaluated at *p* < 0.05 significance level. Relationships between the different parameters analyzed were evaluated by computing the Pearson and Spearman linear correlation coefficients at the *p* < 0.05 confidence level. The homogeneity of variances was determined using Levene’s test.

## 3. Results and Discussion

### 3.1. Effect of Chia Seeds on Physicochemical Characteristics and Antioxidant Properties in Biscuits

Initially, original chia seeds were subjected to different pre-treatments. To obtain ground samples, whole seeds were subjected to a medium grinding level (particle size < 0.8 mm), which allowed them to break up the cell wall but not excessively to avoid the obtaining of flour. Later, defatted ground seeds were obtained from previously sieved ground chia seeds by extraction with hexane. The nutritional composition of wheat flour and chia seeds according to the information declared in the label package is detailed in Table 2.

Compared with wheat flour, chia stands out for its greater content of proteins, simple sugars, fats and fiber and lower content in total carbohydrates, presenting levels in line with results reported by other authors [20]. Fat content was analyzed to check the reduction of the fat content in defatted ground seeds. Thus, fat content decreased from 30% in GS to 8% in DGS. Accordingly, it is assumed that the relative fat reduction quantitatively increased the proportion of remaining compounds in the DGS system.

Seven formulations of biscuits were prepared to maintain the same recipe but partially replace wheat flour with two types of ground chia seeds (defatted and non-defatted) in a range between 5 and 15% of the final weight. Following the directions from the European Commission, chia seed is allowed to be used as an ingredient in baked products at levels lower than 10% [21]. However, percentages up to 15% were tested in order to get more insight into the formation of process contaminants during the biscuit baking process. Although the aim of the present study was not to accurately score the final nutritional value of biscuits, it is assumed that the addition of chia increased the protein, simple sugar, fat and fiber content, thus improving the nutritional profile compared with the control sample. This increase would be even higher when the seeds incorporated were defatted, except for the total fat content, which would increase regarding the control formulation but in less proportion than the non-defatted ground seeds. In this case, the increase in the fat content could be regarded as a positive effect since chia is rich in polyunsaturated fatty acids, mainly ω-3 fatty acids (linolenic acid), considered essential because they cannot be synthesized in the human body and must be obtained through diet [22]. The increase of proteins, fibers and fats with the incorporation of chia in formulations of bakery products has been previously reported by several authors [23,24,25].

Initial ingredients and final biscuits were characterized for different physical and chemical parameters, as included in Table 3 and Table 4, respectively. Table 3 presents the results obtained in the characterization of wheat flour and ground chia seeds (defatted and non-defatted). Several parameters in the raw material, such as moisture, water activity and pH, were evaluated due to their important role in the final properties of the biscuits and also in the development of the chemical reactions during baking. The greatest differences were observed in the moisture content since values were significantly lower in GS (5.54%) and DGS (7.31%), as compared with wheat flour (9.44%). In contrast, although slightly higher in seeds, closer results were observed for the water activity (range: 0.41–0.46) and pH values (Table 3).

The replacement of wheat flour with chia seeds significantly decreased the moisture content and water activity of the biscuits as compared with the control sample (*p* < 0.05), except in samples DGS-5 for moisture and DGS-10 for moisture and water activity in where no differences were observed (Table 4). In DGS biscuits, both moisture and water activity decreased with the higher percentages of seeds, whereas in GS samples, these parameters tended to decrease without following a clear trend linked to the amount of seeds added. After baking, chia biscuits experimented with lower weight loss compared with wheat samples, more markedly with a greater addition of seeds in the formulations. Results agree with those obtained by Coelho et al. [26] in wheat bread formulated with chia flour. These authors demonstrated a minor loss of mass upon baking in formulations with a greater percentage of chia compared to the control sample due to the fiber of chia absorbing a large quantity of water, avoiding its evaporation during the baking process.

Regarding pH, it is assumed that the different ingredients added to the formulations could level out the pH differences observed in the raw materials since no significant differences (*p* > 0.05) were found in biscuits. Results ranged between 7.38 and 7.71, except for DGS-5, in which a significantly higher value was observed (8.05).

**Figure 1:**
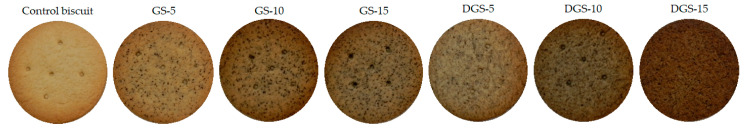
The appearance of wheat-based biscuits (control) with added ground chia seeds (GS) and defatted ground chia seeds (DGS) at 5, 10 and 15%.

Significant differences were observed in the color of the biscuits (Table 4), with all the parameters (*L* *, *a* *, *b* * and E index) decreasing when percentages of chia increased. The color of formulations containing seeds was significantly darker than the control sample (*p* < 0.05), in accordance with results reported in other bakery products enriched with chia [23,24,25,27,28]. It is important to comment that the color parameters of the raw ingredients were very different (Table 3), and therefore, the addition of chia seeds to the biscuits already modified the color of the formulations due to the natural pigments of the seed. Moreover, the evolution of the color could be related to the browning associated with an advanced stage of the Maillard reaction and caramelization [14] in samples with higher content of chia, as will be discussed later. Then, experimental samples exhibited lower values for luminosity (*L* *), ranging from 67.75 (control biscuit) to 55.02 (GS-15) and 48.91 (DGS-15) and for b * parameter, ranging from 24.30 (control biscuit) to 16.34 (GS-15) and 11.42 (DGS-15). DGS samples were the darkest, which could be associated with greater degradation of sugars in this matrix. Regarding the *a* * parameter, the addition of GS increased the values with respect to the control sample, decreasing with greater percentages of seeds added. In contrast, in DGS biscuits, *a* * values decreased with respect to the wheat formulation but increased with the addition of seeds. Changes in the color of the different formulations can also be observed through the ΔE that compares the color of the biscuits with the color of the doughs before baking (ΔE (dough)) and with the color of the control biscuit ΔE (biscuit) (Table 4). Color differences could be related to the chia seeds or to the extent of browning during baking. It is difficult to estimate the contribution of both aspects to the final parameters in the different biscuits. Differences in color and visual appearance of all biscuits can be seen in Figure 1.

The hardness of biscuits, measured as the resistance to breaking, tended to increase in the experimental formulations as compared with the control sample (Table 4). However, the wide variability found in the same batch of biscuits made these differences not to be significant (*p* > 0.05). Changes in hardness by substituting wheat with chia could be associated with the greater contribution of fiber and protein in the experimental samples since it is known that the hardness of the biscuits is related to the presence of fiber and to the interactions between water, starch and protein [29,30]. This fact was already discussed by Lucini Mas et al. [28] in cookies with added defatted chia flour. In agreement with these authors, the lowest hardness values were exhibited in biscuits supplemented with GS, whereas the addition of DGS tended to increase the resistance to breaking. A significant increase in hardness with the increased percentages of chia flour has also been observed in bread samples [24]. Modifications in technological properties such as hardness have also been reported by the addition of soy, corn and bean by-products in gluten-free bakery products [31].

Wheat flour and chia seeds were also characterized by their phenolic acids profile. Except in the case of ferulic acid, chia exhibited a higher presence and content of phenolic acids, highlighting especially the greater levels of chlorogenic and caffeic acids (Table 2), as described in the literature [32,33]. The partial replacement of wheat flour with chia seeds influenced the content of phenolic acids in the biscuits. For most compounds, levels increased with the incorporation of seeds, except for ferulic acid, whose content was higher in the control sample. Despite heat treatment that may cause changes in phenolic profile depending on phenolic acid and baked products [34], in general, higher percentages of chia in biscuits involved higher levels of all the compounds. Values up to 22.89 and 24.57 µg/g for chlorogenic acid, 77.72 and 76.45 µg/g for ferulic acid and 86.40 and 80.17 µg/g for caffeic acid were reached with the addition of 15% GS and DGS, respectively, in line with results reported in biscuits enriched with chia flour [23]. High variability was observed in protocatechuic acid, whereas gallic acid was not identified in any biscuits, which is probably linked to the lower concentrations in raw chia seeds and the degradation of this phenolic acid during the thermal treatment [32].

As expected, due to the high content of phenolic compounds in chia seeds (Table 2), the addition of GS and DGS in the wheat-based biscuit increased the antioxidant properties (Table 5). The TPC values ranged from 305.6 (control sample) to 433.7 (GS-5) and 358.3 µg GAE/g (DGS-5), almost doubling the values with the greater addition of seeds (up to 710.0 and 753.9 µg GAE/g in GS-15 and DGS-15, respectively). Similar results have been reported by Adamczyk et al. [25] in wheat bread, in which the addition of 1% and 5% of ground chia seeds elevated the total phenolic content from 0.09 (control sample) to 0.12 and 0.17 mg GAE/100 g, respectively (results expressed as dry matter). An increase in the TPC when chia flour and seeds were added to the bread and biscuit samples has also been observed by other authors [22,23,27].

The antioxidant capacity assessed by the ABTS assay exhibited a similar trend and an increment was also observed with the addition of chia seeds. In this case, values in control samples (0.68 µmol TEAC/g) changed up to 2.71 and 1.37 µmol TEAC/g in GS-5 and DGS-5, respectively, increasing in an additional 39% and 114% when 15% of these seeds were added to the formulations. Equivalent results have been reported in chips, bread and biscuits enriched with chia flour [22,23,35]. Values obtained in the present study were not those theoretically expected according to the initial results in wheat flour and chia seeds. Therefore, the contribution of other compounds with antioxidant properties generated during the thermal treatment should also be considered [36], the Maillard reaction products among them [17]. An increment in TPC and antioxidant capacity associated with the addition of chia flour to bread and sweet cookies have also been reported [27,28]. In disagreement with these authors, a correlation between total polyphenolic content and antioxidant activity was observed in the present study (ρ = 0.728, *p* = 0.003), probably due to the high contribution of the polyphenols to the antioxidant capacity.

### 3.2. Effect of the Addition of Chia Seeds to Biscuit Formulations on the Content of Process Contaminants

**Figure 2:**
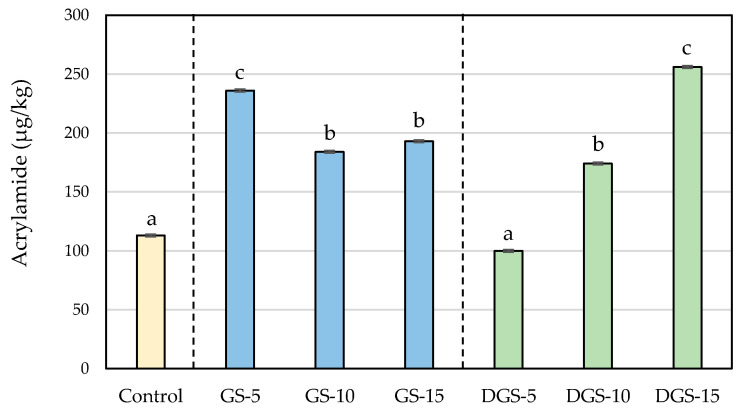
Acrylamide (µg/kg) concentrations in wheat-based biscuits (control) with added ground chia seeds (GS) and defatted ground chia seeds (DGS) at 5, 10 and 15%. Values are mean ± standard deviation. Different letters mean significant differences (*p* < 0.05).

**Figure 3:**
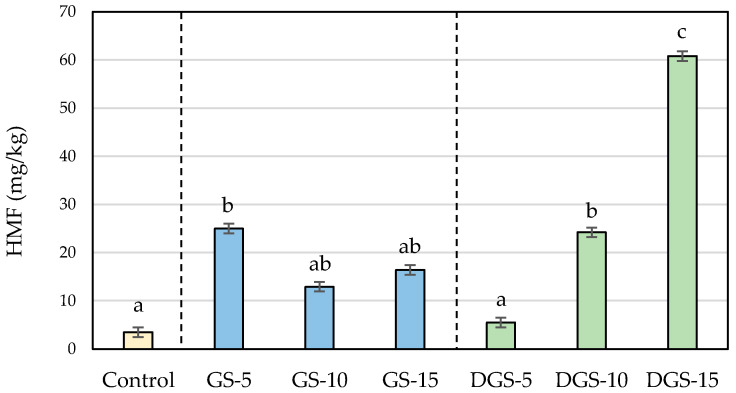
HMF (mg/kg) concentrations in wheat-based biscuits (control) with added ground chia seeds (GS) and defatted ground chia seeds (DGS) at 5, 10 and 15%. Values are mean ± standard deviation. Different letters mean significant differences (*p* < 0.05).

**Figure 4:**
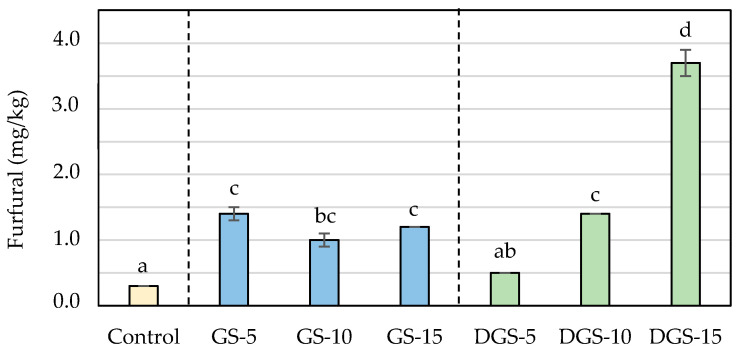
Furfural (mg/kg) concentrations in wheat-based biscuits (control) with added ground chia seeds (GS) and defatted ground chia seeds (DGS) at 5, 10 and 15%. Values are mean ± standard deviation. Different letters mean significant differences (*p* < 0.05).

During baking, high temperatures promote the development of the Maillard reaction and the sugar caramelization and, consequently, the browning and the formation of so-called chemical process contaminants [37]. In the experimental samples, the lower moisture and the higher sugar and protein content of the chia seeds compared to wheat flour make the conditions more favorable for the development of these reactions, leading to obtaining of darker colors in the final product and, in general, higher levels of acrylamide, hydroxymethylfurfural (HMF) and furfural. Levels of process contaminants in the different formulations are presented in Figure 2, Figure 3 and Figure 4. 

The results clearly show that the addition of chia seeds has a strong influence on acrylamide formation in biscuits. The control sample presented levels of acrylamide of 113 µg/kg, which increased significantly in the experimental biscuits except in DGS-5, reaching values higher than 250 µg/kg. In spite of the increase, no biscuits exceeded the benchmark levels reported for this foodstuff in the European Regulation (EU) 2017/2158 (350 µg/kg) [38]. Greater changes were observed in previous studies of this research group in which acrylamide levels in wheat-based biscuits (151 µg/kg) increased until 1188 µg/kg with the addition of 10% of chia flour [23].

Since all the formulations were baked at the same conditions, the higher formation of the contaminant should be related to the levels of precursors in the different recipes. In this regard, chia seeds showed higher content of acrylamide precursors than wheat flour, which would explain the greater acrylamide formation in chia samples. Among precursors, asparagine is the main factor influencing acrylamide formation in bakery products [39]. As expected due to the higher protein content in chia seeds (Table 2), asparagine was significantly higher in chia compared with wheat, with similar content in GS and DGS (Table 3). The values are in line with those reported in literature both for wheat flour and chia seeds [10,39,40], although slight differences could be observed due to common factors such as the genetic basis, growing conditions, time of harvest and postharvest storage conditions largely affect the accumulation of free asparagine in crops, leading to very variable concentrations even for a same food matrix [39]. Despite the same content of free asparagine, the replacement of wheat flour by GS and DGS led to different formations of acrylamide during the baking of biscuits. Moreover, the use of different percentages of chia seeds resulted in different rates of acrylamide formation. When 5% of GS was used, acrylamide levels increased by 109%, reaching a maximum content (236 µg/kg) that decreased up to 184 µg/kg in GS-10 and to 193 µg/kg in GS-15. On the contrary, biscuits with DGS displayed acrylamide concentrations that raised proportionally to the number of seeds added (from 100 µg/kg in DGS-5 to 256 µg/kg in DGS-15), involving increases of 126%. As mentioned before, the relative fat reduction could quantitatively increase the proportion of other compounds in defatted seeds, sugar content among them, promoting the development of the Maillard reaction. Results agree with our previous work, in which acrylamide formation increased faster during the roasting of defatted ground seeds compared with non-defatted samples, likely due to the relatively higher concentration of precursors in the defatted sample [10].

Acrylamide is mainly generated through the Maillard reaction between free asparagine and carbonyl compounds, usually reducing sugars but also reactive carbonyls formed by the thermal oxidation of polyunsaturated fatty acids [41,42,43]. Chia seeds are characterized by their high content of fats (around 30% of their weight), being especially rich in polyunsaturated fatty acids (ω-3 (54–67%) and ω-6 fatty acids (12–21%)) [4,5]. Taking into account that all biscuit formulations included 26 g of sunflower oil, the increase in the percentage of fat in the experimental samples would oscillate between 11.6% (control formulation) and 16.15% for GS-15 or 12.85% for DGS-15. From the results, it could be suggested that degradation products from lipid oxidation did not play an important role as precursors for the formation of acrylamide in GS biscuits, probably due to the slight increase in the fat content but also to the protection of oxidation by antioxidants such as tocopherols presents in chia seeds, which can be efficient in scavenging dicarbonyls formed through the Maillard reaction [44]. In this sense, a significant decrease in the content of tocopherols during thermal treatment has been described in roasted chia [32].

Together with the sugar and asparagine content, the presence of other compounds, such as fiber or phenolic compounds, could also have an influence on acrylamide formation. It is known that dietary fiber reduces the water activity of the product, which could increase the concentration of acrylamide precursors and favor then the Maillard reaction, as has been demonstrated in high-fiber okara flour and in biscuits formulated with chickpea flour [45,46]. However, water activities below 0.6 could decrease the mobility of the reactants, therefore affecting the interaction between them and inhibiting the development of the reaction [47]. In this context, the influence of the fiber in GS and DGS on the different levels of acrylamide formation cannot be discarded. Regarding phenolic compounds, contradictory information has been reported in the literature. Some authors have described that they may inhibit acrylamide formation in heat-treated food [48], and, in contrast, other authors have indicated that compounds such as chlorogenic acid and ferulic acid promote acrylamide formation during heating [49,50]. The possible influence of phenolic compounds on acrylamide generation cannot be ruled out.

The presence of chia seeds also promoted the formation of HMF and furfural in biscuits in ranges similar to those found in biscuits with chia flour [23]. Whereas the control sample presented levels of 3.5 mg/kg for HMF and 0.3 mg/kg for furfural, chia biscuits reached values up to 60.8 mg/kg for HMF and 3.7 mg/kg for furfural (Figure 3 and Figure 4). Similar to acrylamide, the highest concentrations were observed when 5% of ground chia was added to the biscuits, reaching the maximum level (25 mg/kg for HMF and 1.4 mg/kg for furfural) and decreasing with higher incorporation of seeds. On the contrary, furanic compounds in DGS biscuits raised proportionally to the addition of seeds (from 5.5 to 60.8 mg/kg for HMF and from 0.5 to 3.7 mg/kg for furfural, in DGS-5 and DGS-15, respectively), involving increases of more than twelve and fifteen times probably associated with a greater degradation of the sugars in the defatted samples. That degradation, in addition, can give rise to the formation of dicarbonyl compounds, which can act as reactive intermediates of the Maillard reaction boosting the development of the reaction and justifying then the major content of process contaminants [51].

## 4. Conclusions

Although the partial substitution of wheat flour for chia seeds could result in an improvement of the nutritional value and antioxidant properties in bakery products, the formation of other unwanted compounds, such as chemical process contaminants, should also be considered. The replacement of wheat flour with ground chia seeds in formulations of biscuits accelerates the Maillard reaction and caramelization reactions due to the greater precursors content but probably also to the higher dietary fiber in chia, which reduces the moisture and water activity in the recipes. Biscuits with 5% ground chia seeds and 15% defatted ground chia seeds significantly increased the acrylamide levels compared with the control samples (biscuits with 100% wheat flour). Moreover, furanic compounds were increased in all the experimental samples, reaching even levels more than 10 times higher than wheat-based biscuits. The evaluation of the safety parameters mentioned earlier, together with the nutritional enhancement of the recipe, would improve the overall quality and safety of the food, also meeting consumers’ needs and preferences.

## Figures and Tables

**Table 1:** Recipes for the elaboration of wheat-based biscuits (control) with added ground chia seeds (GS) and defatted ground chia seeds (DGS) at 5, 10 and 15%.

Sample	Wheat Flour (g)	Chia Seeds (g)	White Sugar (g)	Distilled Water (mL)	Sunflower Oil (g)	NaHCO_3_ (g)	NH_4_HCO_3_ (g)	Salt (g)
Control	130.0	0	35	30	26	0.8	0.4	1.0
GS-5	118.8	11.2	35	30	26	0.8	0.4	1.0
GS-10	107.7	22.3	35	30	26	0.8	0.4	1.0
GS-15	96.5	33.5	35	30	26	0.8	0.4	1.0
DGS-5	118.8	11.2	35	30	26	0.8	0.4	1.0
DGS-10	107.7	22.3	35	30	26	0.8	0.4	1.0
DGS-15	96.5	33.5	35	30	26	0.8	0.4	1.0

**Table 2:** The nutritional compositions of wheat flour and chia seeds are declared on the label package. Data are expressed per 100 g of sample.

Sample	Wheat Flour	Chia Seeds
Energy (kcal)	350	455
Proteins (g)	10	22.3
Total carbohydrates (g)	73	37.4
simple sugars (g)	0.6	2.7
Total dietary fiber (g)	10	28.4
Fats (g)	1.3	29.5
saturated fats (g)	0.1	2.8
Salt (g)	0.02	0.04

**Table 3:** Characterization of the raw ingredients.

Sample	Wheat Flour	GS	DGS
Moisture (%)	9.44 ± 0.15 ^c^	5.54 ± 0.06 ^a^	7.31 ± 0.05 ^b^
Water activity	0.41 ± 0.01 ^a^	0.43 ± 0.01 ^b^	0.46 ± 0.01 ^b^
pH	6.35 ± 0.04 ^a^	6.59 ± 0.01 ^b^	6.52 ± 0.02 ^b^
Color parameters			
parameter *a* *	−0.32 ± 0.04 ^a^	1.99 ± 0.02 ^c^	1.64 ± 0.05 ^b^
parameter *b* *	9.50 ± 0.54 ^ab^	10.14 ± 0.60 ^b^	8.36 ± 0.36 ^a^
parameter *L* *	84.27 ± 3.15 ^c^	52.85 ± 1.18 ^a^	59.05 ± 2.24 ^b^
E Index	84.81 ± 3.18 ^b^	53.85 ± 1.27 ^a^	59.66 ± 2.27 ^a^
Free asparagine (mg/100 g)	10.17 ± 3.04 ^a^	23.79 ± 0.68 ^b^	23.50 ± 0.53 ^b^
Phenolic acids (µg/g)			
chlorogenic acid	<LOQ	130.25 ± 5.08 ^b^	111.57 ± 2.01 ^a^
*p*-hydroxybenzoic acid	1.31 ± 0.02 ^a^	6.76 ± 0.24 ^b^	7.96 ± 0.07 ^c^
vanillic acid	<LOQ	16.64 ± 0.85 ^a^	21.16 ± 1.15 ^b^
*p*-coumaric acid	5.00 ± 0.48 ^a^	14.40 ± 0.21 ^b^	18.33 ± 0.08 ^c^
ferulic acid	103.16 ± 3.52 ^b^	63.85 ± 0.93 ^a^	73.70 ± 5.61 ^a^
gallic acid	<LOQ	1.41 ± 0.08 ^a^	1.31 ± 0.17 ^a^
protocatechuic acid	<LOQ	16.93 ± 0.15 ^a^	36.69 ± 2.37 ^b^
caffeic acid	3.35 ± 0.16 ^a^	542.58 ± 5.75 ^c^	368.35 ± 5.80 ^b^
HMF (mg/kg)	<LOQ	<LOQ	<LOQ
Furfural (mg/kg)	<LOQ	<LOQ	<LOQ
Acrylamide (µg/kg)	<LOQ	<LOQ	<LOQ
TFC (mg GAE/g)	0.18 ± 0.06 ^a^	2.31 ± 0.04 ^b^	2.24 ± 0.01 ^b^
ABTS (µmol TEAC/g)	1.08 ± 0.02 ^a^	7.09 ± 0.12 ^b^	8.14 ± 0.07 ^c^

GS—Ground chia seeds. DGS—Defatted ground chia seeds. HMF—hydroxymethylfurfural. LOQ—Limit of quantification. TFC—Total Folin Content. GAE—Gallic acid equivalent. ABTS—2,2′-azino-bis (3-ethylbenzothiazoline-6-sulfonic acid. TEAC—Trolox equivalent antioxidant activity. Results are mean ± standard deviation (*n* = 6). Different letters (^a, b, c^) in the same row mean significant differences (*p* < 0.05).

**Table 4:** Physicochemical characterization of biscuits with or without chia seeds.

Sample	Control	GS-5	GS-10	GS-15	DGS-5	DGS-10	DGS-15
Moisture (%)	2.10 ± 0.03 ^c^	1.29 ± 0.03 ^a^	1.36 ± 0.07 ^ab^	1.05 ± 0.13 ^a^	2.34 ± 0.08 ^c^	1.68 ± 0.11 ^b^	1.24 ± 0.05 ^a^
Water activity	0.10 ± 0.00 ^cd^	0.05 ± 0.00 ^a^	0.06 ± 0.00 ^ab^	0.05 ± 0.01 ^a^	0.11 ± 0.00 ^d^	0.08 ± 0.01 ^bc^	0.05 ± 0.00 ^a^
Weight loss (%)	3.06 ± 0.22 ^a^	3.05 ± 0.26 ^a^	2.93 ± 0.33 ^a^	2.80 ± 0.46 ^a^	3.05 ± 0.21 ^a^	2.75 ± 0.70 ^a^	2.74 ± 0.44 ^a^
pH	7.71 ± 0.06 ^a^	7.56 ± 0.02 ^a^	7.55 ± 0.01 ^a^	7.51 ± 0.01 ^a^	8.05 ± 0.16 ^b^	7.68 ± 0.04 ^a^	7.38 ± 0.04 ^a^
Color parameters							
parameter *a* *	5.87 ± 1.84 ^bc^	6.94 ± 1.90 ^c^	5.18 ± 1.90 ^b^	5.05 ± 1.22 ^b^	3.18 ± 1.19 ^a^	4.34 ± 1.19 ^ab^	4.38 ± 0.94 ^ab^
parameter *b* *	24.30 ± 1.56 ^f^	20.23 ± 1.67 ^e^	17.63 ± 1.34 ^d^	16.34 ± 0.99 ^cd^	15.72 ± 1.11 ^c^	13.79 ± 1.04 ^b^	11.42 ± 0.68 ^a^
parameter *L* *	67.75 ± 1.71 ^e^	58.12 ± 4.43 ^d^	57.01 ± 2.47 ^cd^	55.02 ± 2.46 ^bc^	58.77 ± 1.41 ^d^	53.60 ± 1.92 ^b^	48.91 ± 1.53 ^a^
E Index	72.26 ± 1.45 ^e^	61.99 ± 4.40 ^d^	59.95 ± 2.33 ^cd^	57.63 ± 2.41 ^bc^	60.94 ± 1.23 ^d^	55.54 ± 1.81 ^b^	50.43 ± 1.48 ^a^
ΔE (dough)	13.82	15.41	18.20	15.97	11.93	13.14	7.31
ΔE (control)	-	−10.27	−12.31	−14.63	−11.32	−16.72	−21.83
Hardness (N)	67.61 ± 19.91 ^a^	84.13 ± 20.83 ^a^	79.96 ± 18.64 ^a^	86.23 ± 20.63 ^a^	92.11 ± 29.58 ^a^	105.72 ± 19.79 ^a^	96.96 ± 25.04 ^a^

GS—Ground chia seeds. DGS—Defatted ground chia seeds. Results are mean ± standard deviation (*n* = 6). Different letters in the same row mean significant differences (*p* < 0.05). ΔE (dough) compares the E value with its own raw dough. ΔE (control) compares the E value of chia biscuit with biscuit control.

**Table 5:** Phenolic content and antioxidant properties of biscuits with or without chia seeds.

Sample	Control	GS-5	GS-10	GS-15	DGS-5	DGS-10	DGS-15
Phenolic acids (µg/g)							
chlorogenic acid	<LOQ	7.92 ± 0.71 ^a^	16.12 ± 0.26 ^b^	22.89 ± 1.90 ^bc^	8.37 ± 0.74 ^a^	16.50 ± 0.66 ^b^	24.57 ± 1.77 ^c^
PHB	1.06 ± 0.05 ^a^	1.82 ± 0.06 ^bc^	1.90 ± 0.00 ^bc^	2.74 ± 0.24 ^d^	1.50 ± 0.07 ^ab^	2.25 ± 0.19 ^cd^	2.59 ± 0.09 ^d^
vanillic acid	<LOQ	0.55 ± 0.02 ^a^	2.22 ± 0.11 ^b^	3.02 ± 0.26 ^c^	2.74 ± 0.16 ^bc^	3.23 ± 0.03 ^c^	5.91 ± 0.23 ^d^
*p*-Coumaric acid	3.76 ± 0.32 ^a^	4.05 ± 0.02 ^a^	4.91 ± 0.19 ^a^	7.24 ± 0.45 ^b^	4.45 ± 0.04 ^a^	5.89 ± 0.08 ^b^	6.46 ± 0.34 ^b^
ferulic acid	82.26 ± 1.40	62.66 ± 2.43	74.14 ± 1.23	77.72 ± 3.23	79.85 ± 1.20	81.28 ± 2.54	76.45 ± 1.29
gallic acid	<LOQ	<LOQ	<LOQ	<LOQ	<LOQ	<LOQ	<LOQ
PCA	0.89 ± 0.02 ^a^	<LOQ	4.68 ± 0.16 ^b^	1.30 ± 0.12 ^a^	5.04 ± 0.04 ^b^	7.41 ± 0.72 ^c^	9.29 ± 0.03 ^c^
caffeic acid	2.69 ± 0.14 ^a^	29.67 ± 0.94 ^b^	64.00 ± 0.21 ^cd^	86.40 ± 0.22 ^d^	31.87 ± 0.88 ^b^	57.70 ± 6.28 ^c^	80.17 ± 9.37 ^cd^
TFC (mg GAE/g)	0.31 ± 0.03 ^a^	0.43 ± 0.01 ^b^	0.58 ± 0.03 ^c^	0.71 ± 0.02 ^d^	0.37 ± 0.03 ^ab^	0.59 ± 0.02 ^c^	0.75 ± 0.03 ^d^
ABTS (µmol TEAC/g)	0.68 ± 0.02 ^a^	2.71 ± 0.03 ^bc^	3.13 ± 0.23 ^c^	3.76 ± 0.10 ^c^	1.37 ± 0.11 ^ab^	3.27 ± 0.16 ^c^	2.94 ± 0.27 ^bc^

GS—Ground chia seeds. DGS—Defatted ground chia seeds. PHB—*p*-Hydroxibenzoic acid. PCA—Protocatechuic acid. LOQ—Limit of quantification. TFC—Total Folin Content. GAE—Gallic acid equivalent. ABTS—2,2′-azino-bis (3-ethylbenzothiazoline-6-sulfonic acid. TEAC—Trolox equivalent antioxidant activity. Results are mean ± standard deviation (*n* = 6). Different letters in the same row mean significant differences (*p* < 0.05).

## Data Availability

The data that support the findings of this study are available upon reasonable request.
